# Influences of Initial Surface Conditions on Response Characteristics of Amorphous Silicon Films to Nanosecond Laser Irradiation

**DOI:** 10.3390/mi12070807

**Published:** 2021-07-09

**Authors:** Yingming Ren, Zhiyu Zhang

**Affiliations:** 1 Key Laboratory of Optical System Advanced Manufacturing Technology, Changchun Institute of Optics, Fine Mechanics and Physics, Chinese Academy of Sciences, Changchun 130033, China; rymxin@163.com; 2 Center of Materials Science and Optoelectronics Engineering, University of Chinese Academy of Sciences, Beijing 100049, China

**Keywords:** amorphous silicon film, nanosecond pulsed laser irradiation, nano-structure, micro-protrusion

## Abstract

Although laser-produced micro-/nano-structures have been extensively studied, the effects of the initial surface conditions on the formed micro-/nano-structures have rarely been investigated. In this study, through nanosecond pulsed laser irradiation of unpolished and polished amorphous silicon films, entirely different surface characteristics were observed. The effects of laser irradiation parameters, such as repetition frequency, beam overlap ratio, and scanning velocity, on the surface characteristics were investigated, followed by the characterization of surface roughness, energy-dispersive X-ray spectroscopy, and Raman spectroscopy of the irradiated surfaces. For the unpolished surface, novel micro-protrusions were generated after laser irradiation, whereas no such micro-protrusions were formed on the polished surface. The experimental results indicated that the height of the micro-protrusions could be tuned using laser irradiation parameters and that laser irradiation promoted the crystallization of the amorphous silicon film. Moreover, the formation mechanism of the micro-protrusions was linked to fluctuations of the solid–liquid interface caused by continuous laser pulse shocks at higher repetition frequencies. The findings of this study suggest important correlations between the initial surface conditions and micro-/nano-structure formation, which may enhance our fundamental understanding of the formation of micro-/nano-structures.

## 1. Introduction

Amorphous silicon films have been used in photovoltaic solar cells, displays, electronic devices, and imaging optical sensors [1,2,3,4,5,6]. Since the 1990s, laser-induced crystallization of amorphous silicon films has been studied extensively. For example, Huang et al. [7] found that the sharp Raman peak of properly annealed film is similar to that of crystalline silicon formed by the picosecond laser irradiation of amorphous silicon films. Grigoropoulos et al. [8] investigated the liquid–solid interface movement and temperature change of silicon films during excimer laser annealing. These phase transformations are persistent with the recrystallized poly-Si morphologies. In a study to explain the relationship between the excimer laser fluence gradient and the length of lateral grain growth, Moon et al. [9] found that the lateral growth length increases and the directionality of the grains improves as the fluence gradient increases by excimer laser irradiation of amorphous silicon films.

Compared with single-crystal silicon panels, amorphous silicon films are cost-effective and easy to manufacture and can be prepared on a variety of substrate materials, such as metals and ceramics [10,11]. As an attractive material, flexible solar panels are expected to be used on various surfaces to acquire solar energy. To improve the energy absorption ratio of silicon film surfaces, micro-/nano-structures should be fabricated on them. If micro-/nano-structures can be fabricated on an amorphous silicon film, it greatly reduces its production costs, thus promoting industrialization.

In recent years, a large number of investigations have been conducted regarding the formation of micro-/nano-structures on silicon surfaces through laser processing [12,13,14,15,16]. For instance, Her et al. [17] fabricated an array of sharp conical spikes on silicon film surfaces by femtosecond laser irradiation with fluence of 10 kJ/m^2^. Niitsu and Yan [18] produced irregular nanodot structures on the surface of silicon wafers using a nanosecond pulsed laser while eliminating subsurface grinding damage. Kang et al. [19] found that periodic surface structures with a height variation of 14–30 nm formed near the top surface when annealing a 45-nm-thick amorphous silicon thin film on a glass substrate using a Nd:YAG nanosecond laser. Hong et al. [20] achieved nano-dome surface texturing on an amorphous silicon thin film, using a Nd:YVO4 UV laser, resulting in a 200% increase in optical absorption. These nano-dome-like structures were unevenly distributed on the surface. However, these studies were all carried out under specific surface conditions. Therefore, though a huge amount of literature has been published in this field over the last three decades, comprehensively understanding the influences of different initial surface conditions on the response characteristics of amorphous silicon films to nanosecond laser irradiation remains a big challenge.

In this study, we investigated the fundamental response of amorphous silicon films with different initial surface conditions to nanosecond pulsed laser irradiation by studying the effect of laser irradiation parameters on their surface characteristics. A novel periodic micro-protrusion was obtained on an unpolished silicon film surface. Thereafter, we classified the surface roughness, energy-dispersive X-ray spectroscopy, and Raman spectroscopy of the irradiated surfaces, and then analyzed the formation mechanism of this novel micro-protrusion.

## 2. Materials and Methods

### 2.1. Amorphous Silicon Films

In this experiment, an amorphous silicon film with a thickness of 20 μm was coated on the substrate of reaction-bonded silicon carbide—which has a thermal expansion coefficient similar to that of silicon—using a vacuum magnetron sputtering deposition process. The initial surface of the silicon film was polished using a cerium oxide polishing solution to investigate the effects of the surface conditions. The final thickness of the coating after polishing was 16 μm.

### 2.2. Laser Irradiation Conditions

A nanosecond pulsed laser EP30-G8 (Changchun New Industries Optoelectronics Technology Co., Ltd., Changchun, China) was used in the experiment. The laser has a wavelength of 532 nm (green Gaussian beam) with a typical pulse width of 46 ns. Its nominal beam diameter is 80 μm. 

**Figure 1:**
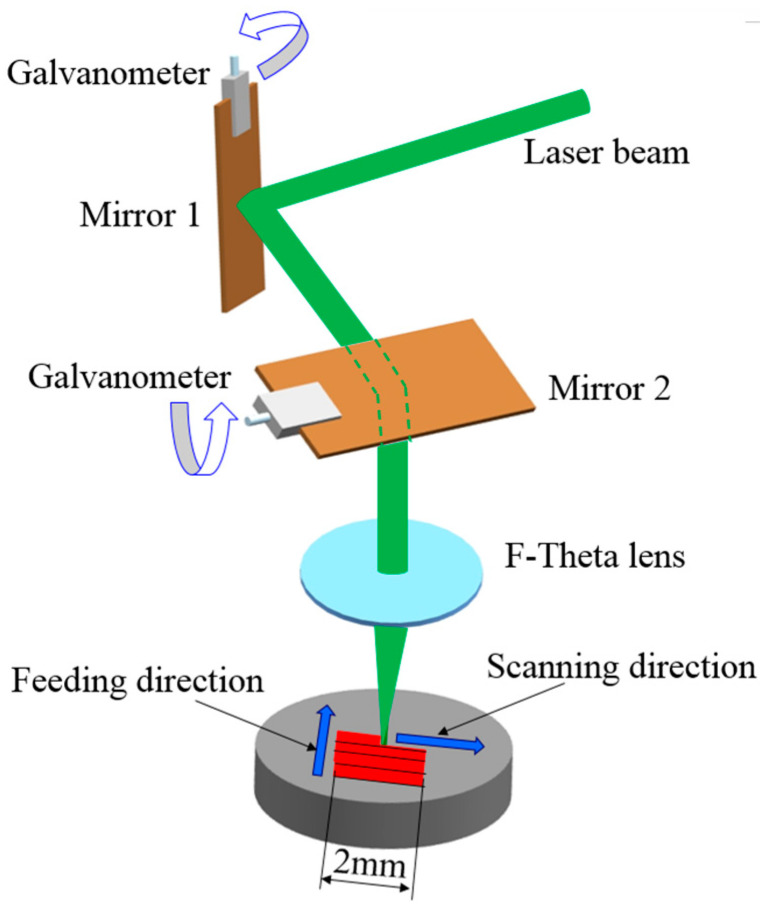
Schematic of the laser system and scanning path.

Figure 1 shows the scanning method of the laser beam during irradiation. The beam was scanned from left to right with a scanning length of 2 mm. After one scan, the galvanometer mirror system directed the laser to the next scan along the feeding direction. Hence, the overlap width between the two scans determined the beam overlap rate.

Table 1 summarizes the laser irradiation conditions. To maintain pulse overlap, the laser repetition frequencies were set from 60 to 160 kHz with a scanning velocity of 50 mm/s. A variety of scanning pitches (10–80 μm) were used, corresponding to beam overlap ratios in the range of 87.5–0%. Average powers of 6, 9, 12, and 15 W were used for laser irradiation. Further, the influence of repetition frequency, average power, and scanning distance were investigated. All laser irradiations were conducted in air at ambient temperature (296–302 K).

### 2.3. Simulation of Laser Irradiation

The silicon film surface absorbs energy from the laser, and the surface temperature increases rapidly. Heat transfer occurs through heat conduction to the material interior, and heat dissipation occurs through radiation and natural convection at the material interface [21,22]. Meanwhile, the evaporation of materials can also lead to energy loss. The heat conduction equation for this process can be expressed as

(1) $$\rho C_{p}\frac{\partial T}{\partial t}+\rho C_{p}\vec{u}\cdot \nabla T+\nabla \left(-k\nabla T\right)=Q,$$

where *ρ* is the density, *C_p_* is the specific heat capacity, *T* is the temperature, *t* is the laser irradiation time, $\vec{u}$ is the velocity field, *k* is the thermal conductivity coefficient, and *Q* is the heat distribution, which can be calculated as follows:

(2) $$Q\left(x,y,z,t\right)=\alpha R\frac{P}{\pi r^{2}}\exp\left(-\frac{2\left(x^{2}+y^{2}\right)}{r^{2}}\right)\exp\left(-\alpha z\right),$$

where *P* is the average laser power, *r* is the radius at 1/e^2^ of the Gaussian laser profile, *R* is the absorptivity of the laser energy, and *α* is the absorption coefficient, which can be defined as

(3) $$\alpha =\frac{4\pi m_{k}}{\lambda },$$

where *m*_k_ is the imaginary part of the refractive index and *λ* is the laser wavelength.

To assess the temperature field produced by laser irradiation on an amorphous silicon film, the finite element method (FEM) was utilized to simulate the heat conduction process. The calculations were performed using COMSOL Multiphysics software. Although the refractive index and absorptivity are functions of temperature, laser energy is primarily absorbed at the material surface, and the temperature distribution can be adequately determined by treating them as constants [23]. The refractive index and absorptivity of amorphous silicon at room temperature were directly employed in this model, which is considered sufficient to study the temperature changes [24].

### 2.4. Measurement and Characterization Methods

A confocal laser scanning microscope (OLYMPUS, OLS4100, Tokyo, Japan; spatial resolution: 100 nm, depth resolution: 1 nm) was used to measure the three-dimensional surface topographies and surface roughness of the laser irradiated regions. Thereafter, the structural changes in the material after laser irradiation were investigated using a micro-Raman spectrometer (LAB-RAM Infinity). Finally, the contents of the elements in the sample before and after laser irradiation were measured using energy-dispersive X-ray spectroscopy (EDX) (EDAX Genesis).

## 3. Results and Discussion

### 3.1. Initial Surface Conditions

**Figure 2:**
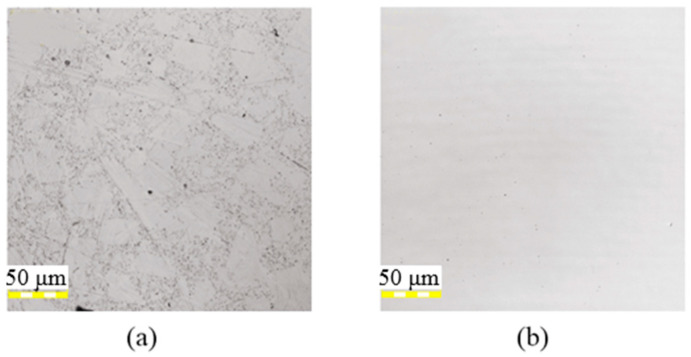
Images of the silicon film surface: (**a**) unpolished surface and (**b**) polished surface.

First, the initial surface conditions were investigated. As shown in Figure 2a, there were some small grain bumps and pitting defects on the unpolished surface. However, as shown in Figure 2b, the polished surface was significantly smoother with only slight abrasive scratches introduced by polishing. The surface roughness of the unpolished and polished surfaces were 12 nm and 1 nm, respectively. Specifically, unpolished and polished silicon films were used as specimens in laser irradiation.

### 3.2. Unpolished Amorphous Silicon Surface

#### 3.2.1. Effects of Repetition Frequency

**Figure 3:**
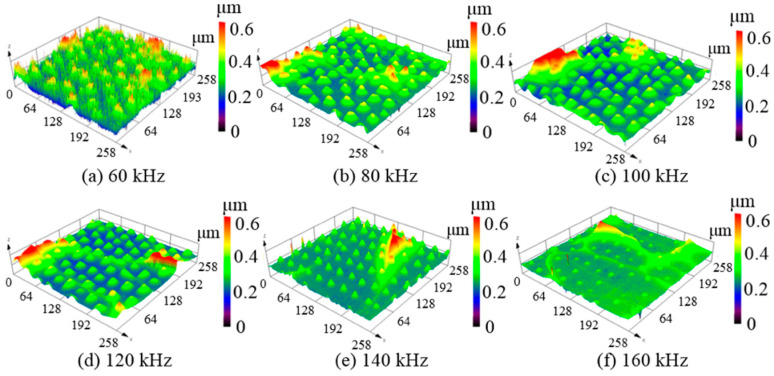
Three-dimensional surface topographies of unpolished silicon film at different repetition frequencies with an average laser power of 9 W and a beam overlap ratio of 50%; (**a**) 60 kHz, (**b**) 80 kHz, (**c**) 100 kHz, (**d**) 120 kHz, (**e**) 140 kHz, (**f**) 160 kHz.

Figure 3 shows the three-dimensional surface topographies after laser irradiation with a repetition frequency of 60–160 kHz. At a low repetition frequency of 60 kHz, there were many cluster structures on the silicon film surface, as shown in Figure 3a. The clustered structures gradually smoothed out to form protrusions as the repetition frequency increased. When the repetition frequency was increased to 80, 100, and 120 kHz, as shown in Figure 3b–d, respectively, the protrusions were irregular with a height variation of 0.1–0.4 μm and various surface damages. As the repetition frequency increased to 140 kHz, periodic micro-protrusions of average height 0.3 μm, as shown in Figure 3e, were obtained. Thus, the height and formation of the protrusions are evidently closely related to the repetition frequency. In addition, for the same average power, the single pulse energy decreased when the repetition frequency was increased. Consequently, the height difference of the protrusions can be associated with a lower ablation rate per pulse for higher repetition frequencies. On the other hand, it should be noted that the average micro-protrusion periodicity was measured as 30 ± 0.05 μm, which does not change with the repetition frequency.

#### 3.2.2. Effects of Laser Beam Overlap Ratio

**Figure 4:**
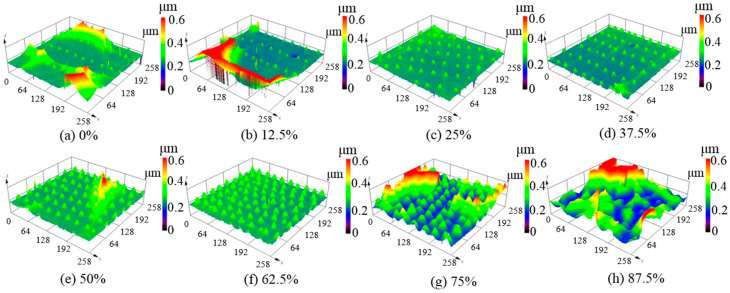
Three-dimensional surface topographies of unpolished silicon film at different laser beam overlap ratios with an average laser power of 9 W and repetition frequency of 140 kHz; (**a**) 0%, (**b**) 12.5%, (**c**) 25%, (**d**) 37.5%, (**e**) 50%, (**f**) 62.5%, (**g**) 75%, (**h**) 87.5%.

The effects of the laser beam overlap ratio were analyzed to obtain more regular micro-protrusions on the silicon film surface. As shown in Figure 4a, when the beam overlap ratio was 0%, the micro-protrusions were small and irregular, with an average height of less than 0.1 μm. When the beam overlap ratio gradually increased, the height micro-protrusions gradually increased and tended to be regular, as shown in Figure 4b–e. The most uniform of them occurred when the beam overlap ratio was 62.5% (Figure 4f) with an average height of 0.3 μm and a surface roughness of Sa 9 nm. Under this condition, the peak power intensity of the laser beam was 4.65 × 10^9^ W/cm^2^.

When the laser beam overlap ratio increased to 75%, the micro-protrusions became slightly larger than those in Figure 4f, as shown in Figure 4g. When the beam overlap ratio increased to 87.5%, almost no convex structures with regular shapes could be observed, as shown in Figure 4h.

#### 3.2.3. Effects of Laser Average Power

**Figure 5:**
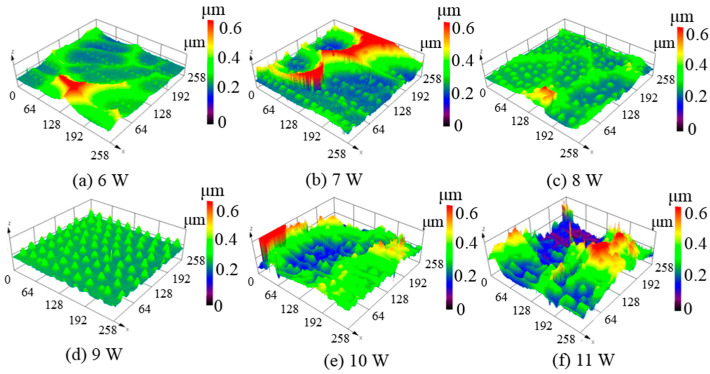
Three-dimensional surface topographies of unpolished silicon film at different laser average powers with a repetition frequency of 140 kHz; (**a**) 6 W, (**b**) 7 W, (**c**) 8 W, (**d**) 9 W, (**e**) 10 W, (**f**) 11 W.

From Figure 4, it can be concluded that relatively uniform micro-protrusions were obtained when the beam overlap ratio was 62.5%. To further verify the effect of laser average power on the micro-protrusions, different laser powers were applied in the experiment. Figure 5 presents the three-dimensional surface topographies of the unpolished amorphous silicon surfaces after laser irradiation. When the average power was 6 W, the micro-protrusions were small, with average height less than 0.1 μm. When the average power gradually increased, the height of the micro-protrusions also gradually increased, as shown in Figure 5b–d. The measured average micro-protrusion periodicity was measured still 30 ± 0.05 μm. However, when the average power increased to 10 W and 11 W, almost no protrusion structures with regular shapes could be observed, as shown in Figure 5e,f. It is assumed that the increase of a single pulse energy caused the melt to splash, resulting in the non-formation of micro-protrusions.

### 3.3. Polished Amorphous Silicon Surface

**Figure 6:**
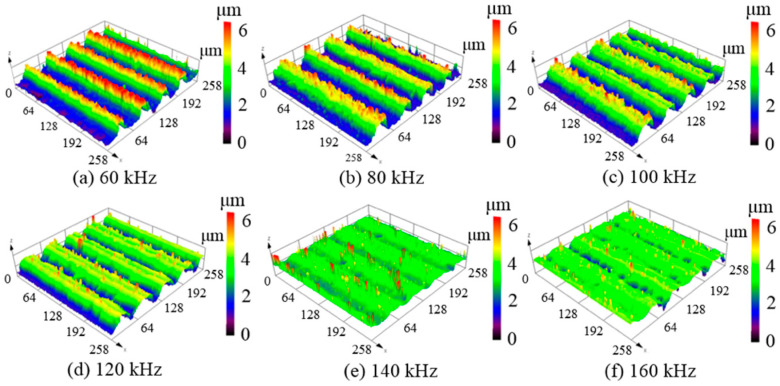
Three-dimensional surface topographies of polished silicon film at different repetition frequencies with an average laser power of 9 W and beam overlap ratio of 50%; (**a**) 60 kHz, (**b**) 80 kHz, (**c**) 100 kHz, (**d**) 120 kHz, (**e**) 140 kHz, (**f**) 160 kHz.

Figure 6 shows the three-dimensional surface topographies of the polished amorphous silicon surfaces after laser irradiation. When the repetition frequency was 60 kHz, the laser scanning path caused distinct grooves with an average depth of 3.2 μm owing to the larger single pulse energy, as shown in Figure 6a. The groove depth gradually decreased with increasing repetition frequency. When the repetition frequencies were increased to 80, 100, and 120 kHz, as shown in Figure 6b–d, the average depths of the grooves on the silicon film surfaces were 2.4, 1.5, and 0.8 μm, respectively. As the repetition frequency increased to 140 kHz, several clustered structures appeared with the disappearance of the relative periodic grooves, as shown in Figure 6e.

**Figure 7:**
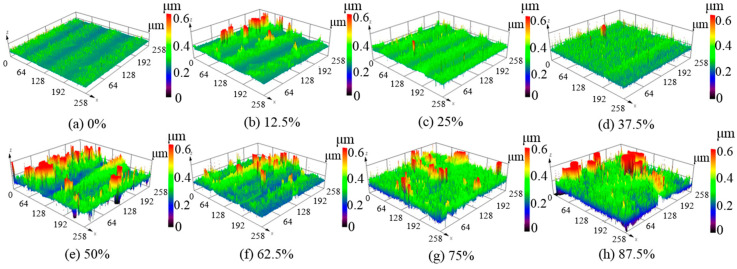
Three-dimensional surface topographies of polished silicon film at different laser beam overlap ratios with an average laser power of 9 W and repetition frequency of 140 kHz; (**a**) 0%, (**b**) 12.5%, (**c**) 25%, (**d**) 37.5%, (**e**) 50%, (**f**) 62.5%, (**g**) 75%, (**h**) 87.5%.

**Figure 8:**
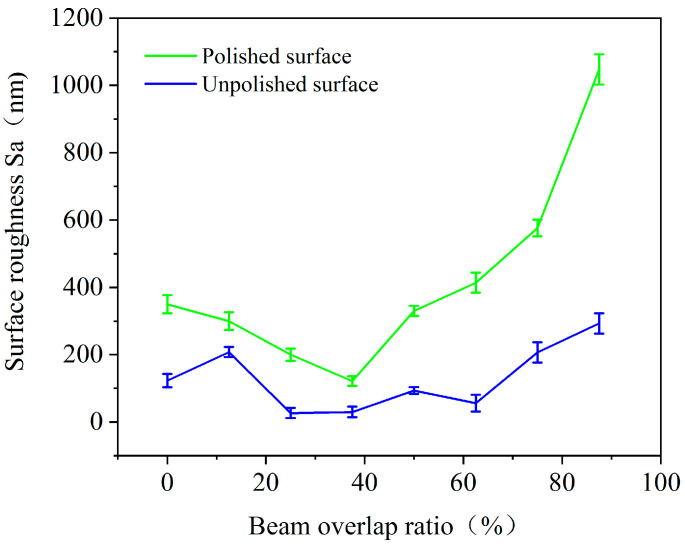
Surface roughness of the irradiated silicon film at various laser beam overlap ratios with an average laser power of 9 W and repetition frequency of 140 kHz.

Figure 7 shows the three-dimensional surface topographies of the polished silicon film after laser irradiation at a repetition frequency of 140 kHz. Despite the same laser parameters as those of Section 3.2.2, completely different surface topographies, stripes, and clusters were formed on the polished silicon film. When the overlap ratio of the scanning trajectory was smaller or larger, severe surface damage was generated, resulting in more surface roughness. As the overlap ratio of the scanning trajectory increased, the surface roughness improved at first before decreasing, reaching its lowest value when the beam overlap ratio was 37.5%, as shown in Figure 7d. Moreover, the surface roughness of the polished silicon film was greater than that of the unpolished silicon film, as shown in Figure 8. However, to a certain extent, the microcracks on the polished surface roughened the surface.

### 3.4. Energy-Dispersive X-ray Spectroscopy Analysis

**Figure 9:**
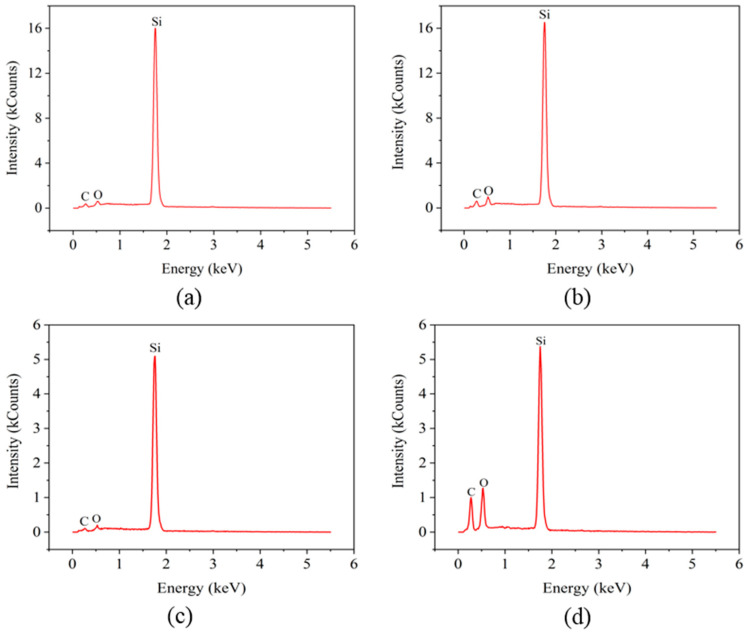
EDX spectra of the unpolished and polished amorphous silicon film surfaces showing the presence of C, O, and Si: (**a**) unirradiated surface of unpolished silicon film, (**b**) irradiated surface of unpolished silicon film, (**c**) unirradiated surface of polished silicon film, and (**d**) irradiated surface of polished silicon film.

To investigate the possible element changes of the silicon film surface caused by laser irradiation, EDX analysis was performed. Figure 9 shows the EDX spectra of the unpolished and polished silicon film surfaces before and after laser irradiation. The major elements on the surface of the silicon film are C, O, and Si. For the unpolished surface, in Figure 9a,b, the content of O increased from 1.48% to 3.97%, indicating that the surface of the silicon film was slightly oxidized after laser irradiation. Compared with the unpolished surface, the polished surface was significantly oxidized after laser irradiation. As shown in Figure 9c,d, the O content increased from 1.45% to 9.16% after laser irradiation. Moreover, the Si content was not affected by laser irradiation, but was reduced by polishing, as shown in Figure 9. Therefore, despite the higher percentage of oxygen on the polished surface after laser irradiation, the amount of oxidation was lower than that of the unpolished surface, as shown in Table 2. This phenomenon indicates that the polished surface absorbs less laser energy due to increased light reflection.

### 3.5. Raman Spectroscopy Analysis

**Figure 10:**
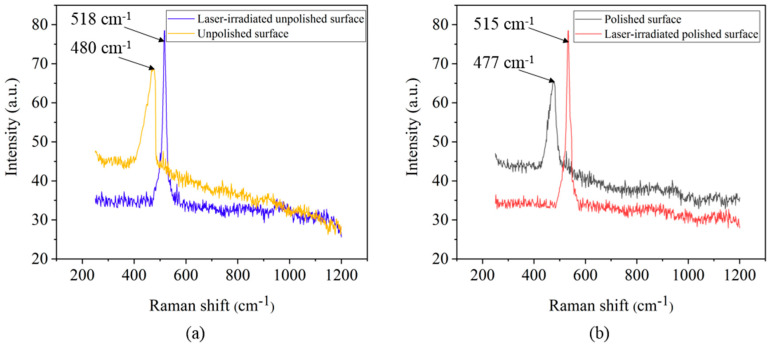
Raman spectra of unpolished and polished silicon film surfaces before and after laser irradiation: (**a**) unpolished surface, (**b**) polished surface.

To investigate the residual stress and possible phase transformation of the silicon film caused by laser irradiation, micro-Raman spectroscopy analysis was performed. Figure 10 shows the normalized Raman spectra of the unpolished and polished silicon film surfaces. The Raman peaks of the unpolished and polished surfaces were 480 cm^−1^ and 477 cm^−1^, respectively. Generally, the Raman peak shift indicates residual stress [18], and full width at half maximum (FWHM) indicates the crystallinity of a material [25]. On the polished surface, the amorphous silicon peak shifted to 477 cm^−1^, indicating that the residual stress layer was removed by the polishing process. Therefore, the material was planarized and homogenized during polishing, leading to a more uniform melting of the material surface during laser irradiation. In addition, no phase transformation was detected on the polished surface based on the Raman spectroscopy results. However, the FWHM of the polished surface (16.41 cm^−1^) was wider than that of the unpolished surface (14.87 cm^−1^), indicating low crystallinity, probably due to dislocations [26].

The crystallinity of the silicon film after laser irradiation was also investigated; and the Raman peaks of amorphous silicon and single-crystal silicon were observed at 470 cm^−1^ and 521 cm^−1^, respectively. As shown in Figure 10, the Raman peaks of the unpolished and polished surfaces after laser irradiation shifted to 518 cm^−1^ and 515 cm^−1^, respectively. Furthermore, the FWHM of the unpolished and polished surfaces after laser irradiation became 8.68 cm^−1^ and 10.35 cm^−1^, respectively. Accordingly, for unpolished and polished surfaces, the Raman peaks increased and the FWHM became narrower after laser irradiation. The results show that amorphous silicon undergoes a crystalline transition during laser irradiation [27].

### 3.6. Formation Mechanism of Micro-Protrusions

**Figure 11:**
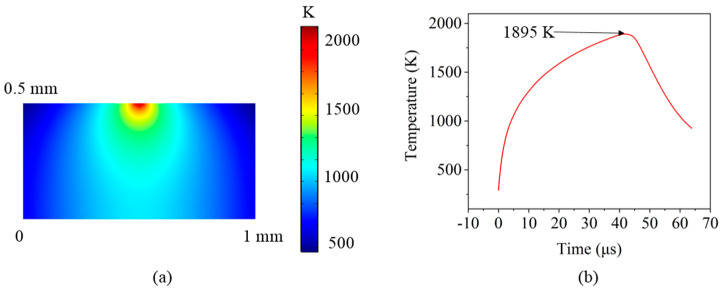
(**a**) Temperature distributions and (**b**) changes of the sample under 4.65 × 10^9^ W/cm^2^ during a laser pulse.

Figure 11 shows the FEM-simulated temperature distributions and changes of the silicon film surface during laser pulse irradiation. Owing to the Gaussian energy distribution of the laser beam, the beam energy gradually attenuated from the center to the surroundings [28], resulting in uneven melting of the surface layer. As shown in Figure 11a, the surface temperature sharply increased, with the high temperature zone extending rapidly downward into the bulk region. The maximum melting temperature was located at the centre of the beam and gradually decreased in the surrounding regions. The FEM simulation results demonstrate that the maximum surface temperature of the silicon film reached 1895 K during laser pulse irradiation, where the temperature was higher than the melting point of amorphous silicon (1420 K), as shown in Figure 11b. Conversely, after the surface of the silicon film rose to its highest temperature, it quickly fell below its melting temperature within 14 μs.

As mentioned in Section 3.2.1, the repetition frequency was significantly related to the formation of micro-protrusions. When the repetition frequency was higher than 60 kHz, the pulse interval time was less than 1.67 × 10^−5^ s. In such an extremely short period of time, the melting caused by the first pulse had not yet been completed, and the second pulse continued to impact the same region. The fluctuations in the solid–liquid interfaces were caused by continuous pulse shocks.

**Figure 12:**
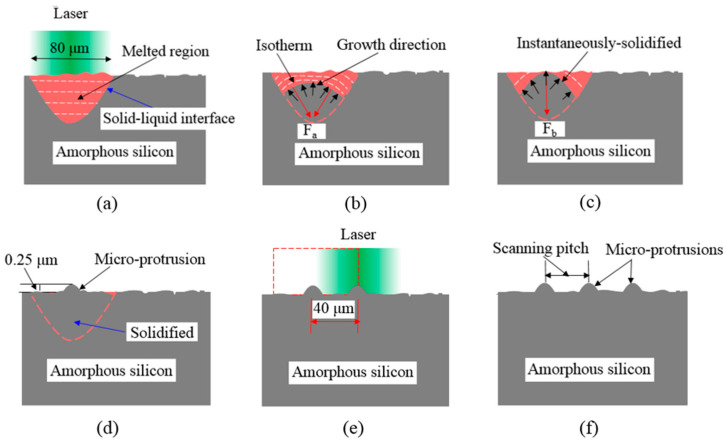
Schematic diagrams of micro-protrusion growth due to solid-liquid interface instability; (**a**) The laser molten pool, (**b**) The instantaneous solidification, (**c**) The instantaneous solidification growth, (**d**) The micro-protrusion formation, (**e**) Two rows of micro-protrusion formed (**f**) Three rows of micro-protrusion formed.

Figure 12 shows schematic diagrams of the micro-protrusion growth. Generally, melting starts at the solid–liquid interface [29]. As shown in Figure 12a, the surface layer melted (liquified) because the surface temperature sharply increased above its melting temperature when laser irradiation was applied to the silicon film surface [30]. The solid–liquid interface of the silicon film and isotherms of the temperature are presented in Figure 12b.

The solid–liquid interface was protuberated in the center region owing to fluctuations created by continuous pulse shocks. The isotherms were partially compressed by the interface with a larger temperature gradient, generating a compressive force F_a_, which promoted the growth of micro-protrusions [18]. Owing to the temperature profile characteristics at the solid–liquid interface, the protrusions grew with the solid–liquid interface fluctuations under compression force F_a_, as shown schematically in Figure 12b. When the protrusions increased in size, the top of the protrusions under the action of surface tension F_b_ became camber-shaped, as shown in Figure 12c [31,32]. Finally, the micro-protrusions formed on the upper surface through the repetitive growth and rounding process, as shown in Figure 12f.

The experimental results indicate that the periodic micro-protrusions were distributed along the beam scanning path, whose height could be determined by the scanning pitch and laser average power, as shown in Figure 4. It is assumed that the altered depth of the molten material between adjacent tracks led to a change in the height of the protrusion structures. Compared with other studies [33,34], we created micron-height regular micro-protrusions owing to the utilization of an ideal surface roughness. This is completely different from the two-dimensional nanodot structures produced on a stainless steel surface by cross-scanning the laser beam [35].

### 3.7. Characteristics of the Polished Surface

**Figure 13:**
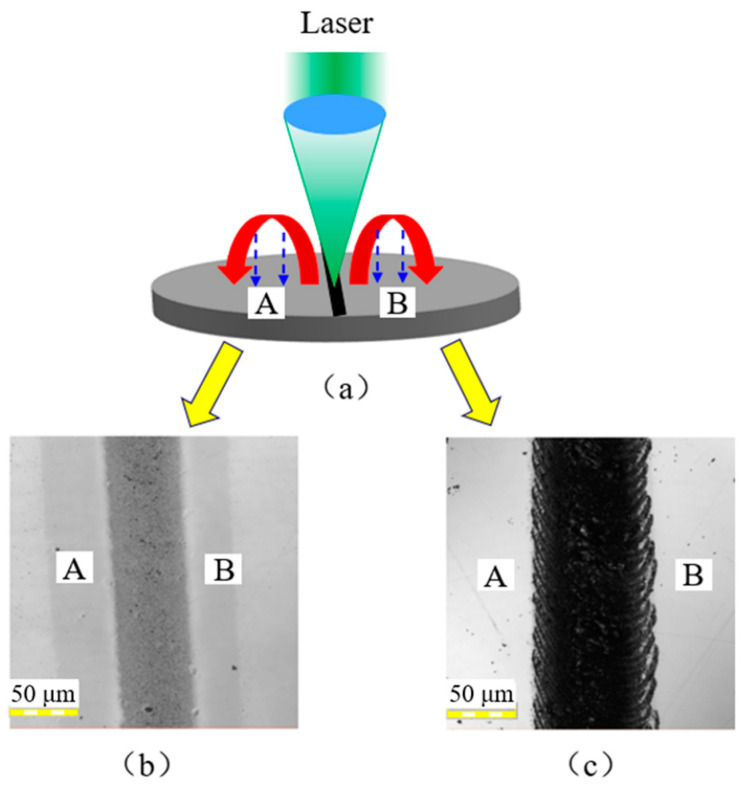
(**a**) Schematic diagram of pulsed laser irradiation polished silicon film surface; (**b**) Microscope image of a single line melted on polished silicon film with an average laser power of 9 W and repetition frequency of 140 kHz; (**c**) Microscope image of a single line ablated on polished silicon film surface with an average laser power of 15 W and repetition frequency of 140 kHz.

Figure 13 shows schematic diagrams of the pulsed laser single track scanning silicon film surface. After polishing, the surface of the silicon film was removed up to a thickness of about 4 μm and a smoother surface was obtained. The residual stress layer generated during the coating process was removed by the polishing process, as shown in Figure 10. Due to the polish-induced surface planarization and homogenization, the surface of the silicon film was uniformly melted after laser irradiation with an average laser power 9 W, as shown in Figure 13b. As there were no large fluctuations and temperature gradients at the solid–liquid interface, micro-protrusions were difficult to produce on the polished silicon film surface through laser irradiation, as shown in Figure 7. On the other hand, there are laser-affected areas on both sides of the melting track due to the Gaussian energy distribution of the laser beam. As shown in Figure 13b, when the average power was increased to 15 W, the silicon film surface was ablated under a high laser fluence. As can be seen from the graph, limited nanoparticles can be observed in regions A and B. They are Si atoms from the groove anisotropic ejection owing to laser fluence being greater than the ablation threshold of the silicon film surface [36]. This also explains the phenomenon of grooves on the polished surface in Figure 6b–d.

### 3.8. Surface Defects Analysis

**Figure 14:**
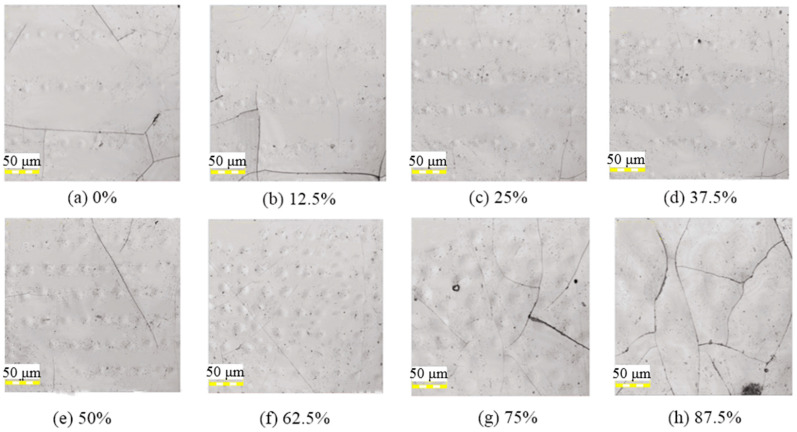
Images of unpolished surface irradiated at different laser beam overlap ratios with an average laser power of 9 W, repetition frequency of 140 kHz, and laser scanning velocity of 50 mm/s; (**a**) 0%, (**b**) 12.5%, (**c**) 25%, (**d**) 37.5%, (**e**) 50%, (**f**) 62.5%, (**g**) 75%, (**h**) 87.5%.

Figure 14 shows the surface topographies of the unpolished surface after laser irradiation. Along with the increasing beam overlap ratio, microcracks tended to decrease first before increasing, as shown in Figure 14a–h. Figure 14f shows that the cracks almost disappeared when the beam overlap ratio was increased to 62.5%. However, when the beam overlap ratios were continuously increased to 75% and 87.5%, numerous cracks were generated on the surface. The open cracks increased the surface roughness and seriously affected the formation of periodic micro-protrusions. In addition, microstructural changes, attachment, nano-sized pores, and bumps were formed on the silicon film surface after laser irradiation.

The generation of microcracks was related to the surface stress induced by the vacuum magnetron sputtering deposition process. At low peak power intensities, thermal expansion resulted in the internal microcracks opening rather than melting the material layer [37], which appeared directly on the silicon film surface, as shown in Figure 14. As the beam overlap ratio increased to 62.5%, as shown in Figure 14f, the cracks were reduced (or even disappeared) owing to the increased melting of the surface layer. However, when the beam overlap ratios were continuously increased to 75% and 87.5%, the microcracks increased again, which may have been caused by the violent pulse shocks, as shown in Figure 14g,h.

**Figure 15:**
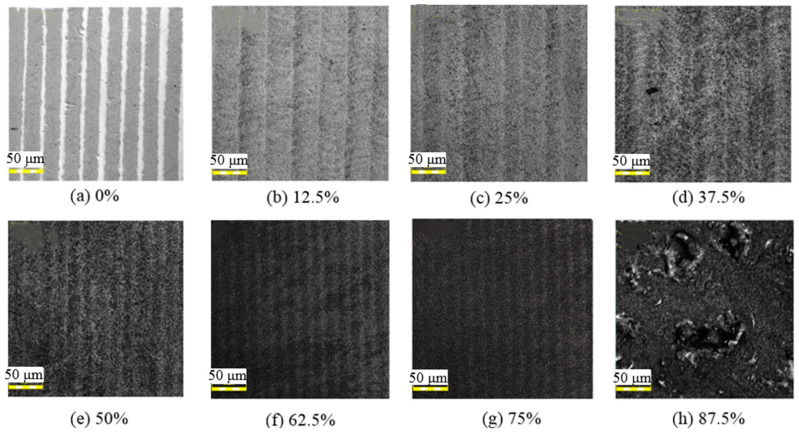
Images of polished surface irradiated at different laser beam overlap ratios with an average laser power of 9 W, repetition frequency of 140 kHz, and laser scanning velocity of 50 mm/s; (**a**) 0%, (**b**) 12.5%, (**c**) 25%, (**d**) 37.5%, (**e**) 50%, (**f**) 62.5%, (**g**) 75%, (**h**) 87.5%.

Figure 15 shows the surface topographies of the polished surface after laser irradiation. In Figure 15a, there are some clear melting tracks due to the lack of overlap between the two beam scans. As the beam overlap ratio increased, the melting tracks gradually covered the entire surface. When the beam overlap ratio was increased to 12.5% and 25%, regular textured structures corresponding to the laser scanning trajectory appeared on the entire surface, as shown in Figure 15b,c. When the beam overlap ratio was 87.5%, as shown in Figure 15h, serious surface defects, such as slags, welds, and pits, were generated on the surface.

## 4. Conclusions

In this study, the effects of initial surface conditions on the surface topography formation of amorphous silicon films irradiated by a nanosecond laser were investigated. The following conclusions were drawn:

1. A completely different phenomenon appeared after laser irradiation of the unpolished and polished silicon film surfaces. There were mainly cracks, attachments, nano-sized pores, and bumps on the irradiated unpolished surface, while there were mainly corrosive pitting, debris, and slag on the irradiated polished surface because the surface stress layer was removed by the polishing process.
2. Periodic micro-protrusions could be obtained on the unpolished amorphous silicon surface, but not on the polished surface. The main irradiation parameters affecting the formation of micro-protrusions were the laser beam overlap ratio, repetition frequency, and average power, among which the most crucial parameter was the repetition frequency.
3. At a repetition frequency of 140 kHz under a peak power intensity of 4.65 × 10^9^ W/cm^2^, periodic micro-protrusions with an average height of 0.3 μm and the same roughness as the initial surface could be obtained.
4. The formation of micro-protrusions was related to the fluctuations of the solid–liquid interface caused by continuous laser pulse shocks during laser irradiation. Owing to the instantaneous heating-cooling cycle (10^7^ K/s) between laser pulses, there was insufficient time for the surface tension to flatten the instantaneously solidified local regions on the solid–liquid interface. This type of heating-cooling cycle enabled top-down melting and bottom-up protrusion growth.
5. The non-formation of micro-protrusions was probably due to polish-induced surface planarization and homogenization. Raman analysis showed that the amorphous silicon film crystallized during laser irradiation. The removal of surface stress and reduction in surface roughness were both verified.

These results indicate entirely different responses of the unpolished and polished amorphous silicon surfaces to nanosecond pulsed laser irradiation. The findings of this study will contribute to understanding the formation mechanism of micro-protrusions from the viewpoint of initial surface conditions and should be appealing for use in potential applications, such as flexible solar panels, wearable electronic devices, and superhydrophobic/hydrophilic surfaces.

## Figures and Tables

**Table 1:** Laser irradiation conditions.

Items	Parameters
Laser type	Nanosecond pulsed laser
Wavelength	532 nm
Pulse width	46 ns
Laser average power	6−15 W
Repetition frequency	30−160 kHz
Scanning pitch	10−80 μm
Scanning velocity	50 mm/s
Beam overlap ratio	0−87.5%
Irradiation region	2 × 2 mm^2^
Environment	Air at ambient temperature

**Table 2:** Changes in the oxygen (O) content before and after laser irradiation on the unpolished and polished surface.

O Element	Total (kCounts)	Initial Value (kCounts)	Final Value (kCounts)	Increment (kCounts)
Unpolished surface	17.03	0.25	0.68	0.43
Polished surface	5.20	0.48	0.08	0.40

## References

[1] Nishimoto T., Takai M., Miyahara H., Kondo M., Matsuda A. (2002). Amorphous silicon solar cells deposited at high growth rate. J. Non-Cryst. Solids.

[2] Madan A. (2007). Flexible displays and stable high efficiency four terminal solar cells using thin film silicon technology. Adv. Manuf. Process..

[3] Derkacs D., Lim S.H., Matheu P., Mar W., Yu E.T. (2006). Improved performance of amorphous silicon solar cells via scattering from surface plasmon polaritons in nearbymetallic nanoparticles. Appl. Phys. Lett..

[4] He L., Xu J., Dekai Z., Yang Q.H., Li L.O. (2018). Potential application of functional micro-nano structures in petroleum. Pet. Explor. Dev..

[5] Xiang T., Hou J.W., Xie H., Liu X., Gong T., Zhou S.B. (2020). Biomimetic micro/nano structures for biomedical applications. Nano Today.

[6] Huang H., Jiang M.Q., Yan J.W. (2018). Softening of Zr-based metallic glass induced by nanosecond pulsed laser irradiation. J. Alloy. Compd..

[7] Huang C.R., Lee M.C., Chang Y.S. (1990). Optical and Raman correlation of laser recrystallised and quenched amorphous silicon film: A microprobe study. J. Phys. D Appl. Phys..

[8] Grigoropoulos C.P., Moon S., Lee M. (1999). Thermal transport in melting and recrystallization of amorphous and polycrystalline Si thin films. Appl. Phys. A.

[9] Lee M., Moon S., Hatano M. (2000). Relationship between fluence gradient and lateral grain growth in spatially controlled excimer laser crystallization of amorphous silicon films. J. Appl. Phys..

[10] Soederstroem T., Haug F.J., Terrazzoni-Daudrix V., Ballif C. (2008). Optimization of amorphous silicon thin film solar cells for flexible photovoltaics. J. Appl. Phys..

[11] Huang H., Lu L., Wang J.W., Jie Y., Leung S.F., Di C., Chen X., Shen G. (2013). Performance enhancement of thin-film amorphous silicon solar cells with low cost nanodent plasmonic substrates. Energy Environ. Sci..

[12] Jian C., Liu C.S., Shang S., Perrie W., Dearden G., Watkins K. (2013). A review of ultrafast laser materials micromachining. Opt. Laser Technol..

[13] Wang Y., Liu W., Xin W., Zou T., Guo C. (2020). Back-Reflected Performance-Enhanced Flexible Perovskite Photodetectors through Substrate Texturing with Femtosecond Laser. ACS Appl. Mater. Interfaces.

[14] Malinauskas M., Zukauskas A., Hasegawa S., Hayasaki Y., Mizeikis V., Buividas R., Juodkazis S. (2016). Ultrafast laser processing of materials: From science to industry. Light Sci. Appl..

[15] Svrcek V., Mariotti D., Cvelbar U., Filipi G., Lozach M., Mcdonald C., Tayagaki T., Matsubara K. (2016). Environmentally friendly processing technology for engineering silicon nanocrystals in water with laser pulses. J. Phys. Chem. C.

[16] Borisov R.A., Dorojkina G.N., Koroteev N.I. (1998). Femtosecond two-photon photopolymerization: A method to fabricate optical photonic crystals with controllable parameters. Laser Phys..

[17] Her T.H., Finlay R.J., Wu C. (1998). Microstructuring of silicon with femtosecond laser pulses. Appl. Phys. Lett..

[18] Niitsu K., Yan J.W. (2019). Effects of deep subsurface damages on surface nanostructure formation in laser recovery of grinded single-crystal silicon wafers. Precis. Eng..

[19] Kang M.J., Park T.S., Kim M., Hwang E.S., Kim S.H., Shin S.T., Cheong B.H. (2019). Periodic surface texturing of amorphous-Si thin film irradiated by UV nanosecond laser. Opt. Mater. Express.

[20] Hong L., Wang X., Rusli, Wang H., Zheng H., Yu H. (2012). Crystallization and surface texturing of amorphous-Si induced by UV laser for photovoltaic application. J. Appl. Phys..

[21] Jiao L.G., Li J.S., Yuan C., Jiang H.M., Zhao G.M. (2014). Irradiation effects of continuous laser on liquid tank: A natural convection study. Int. J. Heat Mass Transf..

[22] Stephan K., Abdelsalam M. (1980). Heat-transfer correlations for natural convection boiling. Int. J. Heat Mass Transf..

[23] Kumar N., Dash S., Tyagi A.K., Raj B. (2010). Dynamics of plasma expansion in the pulsed laser material interaction. Sadhana.

[24] Ghosh G. (1995). Temperature dispersion of refractive indices in crystalline and amorphous silicon. Appl. Phys. Lett..

[25] Xu Z.W., Zhong D., He Y. (2018). Topic Review: Application of Raman Spectroscopy Characterization in Micro/Nano-Machining. Micromachines.

[26] Kundu P.P., Tripathy D.K., Banerjee S. (1996). Studies on the miscibility of blends of polychloroprene and poly (ethylene-methyl acrylate) copolymer. Polymer.

[27] Bissett M.A., Izumida W., Saito R., Ago H. (2012). Effect of domain boundaries on the Raman spectra of mechanically strained graphene. ACS Nano.

[28] Chen G., Bi J. (2016). Analytical solutions for three-dimensional modeling of temperature rise inside solid material induced by laser irradiation. Optik.

[29] Xu X., Grigoropoulos C.P., Russo R.E. (1994). Measurement of solid–liquid interface temperature during pulsed excimer laser melting of polycrystalline silicon films. Appl. Phys. Lett..

[30] Hatano M., Moon S., Lee M. (2000). Excimer laser-induced temperature field in melting and resolidification of silicon thin films. J. Appl. Phys..

[31] Ikegami M., Weaver T.E., Grant S.N., Whitsett J.A. (2009). Pulmonary surfactant surface tension influences alveolar capillary shape and oxygenation. Am. J. Respir. Cell Mol. Biol..

[32] Liu N., Huang W.M., Phee S.J., Tong T.H. (2008). The formation of micro-protrusions atop a thermo-responsive shape memory polymer. Smart Mater. Struct..

[33] Kushnira V.V., Rubashkinaa M.V., Svetlichnyia A.M. (2012). The formation of submicron structures on the surface of amorphous silicon films by nanosecond pulsed radiation of a laser line generator. Nanotechnol. Russia.

[34] Franta B., Mazur E., Sundaram S.K. (2017). Ultrafast laser processing of silicon for photovoltaics. Int. Mater. Rev..

[35] Kobayashi T., Yan J.W. (2017). Generating nanodot structures on stainless-steel surfaces by cross scanning of a picosecond pulsed laser. Nanomanuf. Metrol..

[36] Xu K., Zhang C.T., Zhou R., Ji R., Hong M.H. (2016). Hybrid micro/nano-structure formation by angular laser texturing of Si surface for surface enhanced Raman scattering. Opt. Expres..

[37] Huang H., Qian Y.F., Wang C., Yan J.W. (2020). Laser induced micro-cracking of Zr-based metallic glass using 10^11^ W/m^2^ nano-pulses. Mater. Today Commun..

